# The ability of orthodontists and oral/maxillofacial surgeons to predict eruption of lower third molar

**DOI:** 10.1186/s40510-016-0134-0

**Published:** 2016-07-11

**Authors:** Aline do Carmo Bastos, Joelma Bezerra de Oliveira, Karina Flexa Ribeiro Mello, Patrícia Botelho Leão, Flavia Artese, David Normando

**Affiliations:** Department of Orthodontics, Brazilian Association of Dentistry, Trav. Marques de Herval, 2298, Belém, Pará 66.087-320 Brazil; Department of Orthodontics, Rio de Janeiro State University, Boulevard 28 de Setembro, 157, Rio de Janeiro, RJ CEP: 20551-030 Brazil; Department of Orthodontics, Federal University of Pará, Augusto Corrêa St., number 1, College of Dentistry, Belém, Pará 66.075-110 Brazil

**Keywords:** Third molar, Impaction, Extraction, Dental eruption

## Abstract

**Background:**

The aim of this study was to evaluate the ability of oral/maxillofacial surgeons (OMFSs) and orthodontists to predict third molar eruption by examining a simple panoramic radiograph in cases where full spontaneous eruption occurred.

**Methods:**

Panoramic radiographs of 17 patients, 13–16 years of age, were obtained just after orthodontic treatment (T1), when the third molars were intraosseous. The radiographs at T1 were presented to 28 OMFSs and 28 orthodontists—who were asked to give a prognosis for the lower third molars on both sides (*n* = 34). The full spontaneous eruption of all third molars was clinically observed when patients were older than 18 years (T2). These teeth were clinically asymptomatic at T1 and T2.

**Results:**

OMFSs decided by extractions in 49.6 % of cases while orthodontists in 37.8 % (*p* < 0.001), when the radiographs were examined at T1. Agreement between OMFSs and orthodontists was excellent (Kappa = 0.76, *p* < 0.0001), as well as intragroup agreement for both OMFSs (Kappa = 0.83) and orthodontists (Kappa = 0.96).

**Conclusions:**

Despite a remarkable agreement for third molar prognosis, orthodontists and OMFSs were unable to predict lower third molar eruption by examining a simple panoramic radiograph. Both indicated extractions of a considerable number of spontaneously erupted asymptomatic teeth.

## Background

Third molar extraction is one of the most frequent procedures in oral surgery. Ten million teeth are extracted from approximately five million individuals every year in the USA [1]. The reason for third molar removal include the risk of impaction associated with caries, pericoronitis, periodontal defects in the distal surface of second molars, odontogenic cysts, and dental crowding [1–5]. A prospective study showed that general dentists recommended removal of third molars in 59 % of participants, mainly to prevent future problems or because a third molar had an unfavorable orientation or was unlikely to erupt [6]. A recent systematic review evaluated the prevalence of third molar impaction worldwide based on radiographic examination. Worldwide impaction prevalence was found to be 24.40 % (95 % CI, 18.97 to 30.80 %), which is much smaller than the percentage that undergoes clinical treatment for M3 problems [7].

The ideal moment to determine whether or not to remove third molars is also under debate, since impaction prediction has not been scientifically proven. Moreover, it is a daunting task to predict this biological condition with any degree of reliability [8]. Systematic reviews have reported that there is no evidence to support or refute prophylactic removal of asymptomatic impacted third molars, even in adults [9, 10]. The scientific evidence contraindicates the prophylactic removal of third molars in order to prevent late lower anterior crowding [9, 10]. However, in comparing the opinion of orthodontists and oral/maxillofacial surgeons (OMFSs), it became clear that both indicate prophylactic removal of third molars to prevent crowding [11, 12].

Although reports suggest that the predictive power of third molar eruption is low [3] and impacted third molars that remain static, with no changes in position or angulation over time are rare [13], professionals are still highly prone to indicate the extraction of these teeth, often in early adolescence. This study aimed to evaluate the ability of OMFSs, and orthodontists to predict third molar eruption by examining a simple panoramic radiograph in cases where the physiological eruption of third molars was known to have occurred.

## Methods

This study was approved under #498024 by the ethics committee for health science Institute of the Federal University of Pará. The sample included panoramic radiographs of 17 patients at the end of orthodontic treatment obtained from the databases of one private practice (D.N.). Inclusion criteria comprised individuals aged between 13 and 16 years, of both genders, treated without extractions and whose third molars erupted spontaneously years later (mean 6.4 years). All third molars were clinically asymptomatic at T1 and T2. Cases of agenesis, tooth loss, or tooth extractions for orthodontic purposes were excluded from the sample. Patients completed the orthodontic treatment between the years 2005 and 2010 (T1) and were reassessed between 2009 and 2014 (T2).

Fifty-six specialists—28 OMFSs and 28 orthodontists—were asked to provide, based on end-of-treatment panoramic radiographs (T1), their prognosis for the mandibular third molars present in those radiographs (*n* = 34). These specialists were enrolled by voluntary response. The panoramic radiographs taken at the end of orthodontic treatment was randomly presented to each evaluator. Patient’s age and sex were identified. The questionnaire included the following question: How would you approach the right and left lower third molars? Patient A’s radiograph was duplicated in radiograph E in order to evaluate method error, totaling 18 radiographic and 36 third molar evaluations.

The data collected were subjected to statistical analysis, at *p* < 0.05, using BioEstat 5.3 software (Mamirauá Maintainable Development Institute, Belém, Pará, Brazil).

To evaluate intergroup and intragroup agreement, Kappa statistical analysis was used. Chi-square test was used to compare the distribution of responses between orthodontists and oral/maxillofacial surgeons.

## Results

Diagnostic agreement between orthodontists and OMFSs was considered excellent for all patients, with a Kappa value of 0.76 (*p* < 0.0001, Table 1). Intragroup agreement proved excellent for both oral/maxillofacial surgeons (Kappa = 0.83, *p* < 0.0001) and orthodontists (Kappa = 0.96, *p* < 0.0001). Response unanimity was observed in two cases. One hundred percent of the OMFSs indicated extractions for both molars in patient P, while all orthodontists indicated monitoring for patient B.

**Table 1:** Frequency of diagnostic response regarding extraction, monitoring or other alternatives for lower third molars given by orthodontists (n = 28) and oral/maxillofacial surgeons (OMFSs) (n = 28). Frequency distribution was evaluated by chi-square (χ²) and agreement between evaluators by Kappa test

	Orthodontists	OMFSs	Kappa (*p* value)
Extraction	360 (37.8 %)	472 (49.6 %)	0.76 (<0.0001)
Monitoring	586 (61.6 %)	478 (50.2 %)	
Others	6 (0.6 %)	2 (0.2 %)	
Total	952 (100 %)	952 (100 %)	
Ortho × OMFSs (*x* ^2^)	25.56 (*p* < 0.0001), power 99 %		

**Fig. 1:**
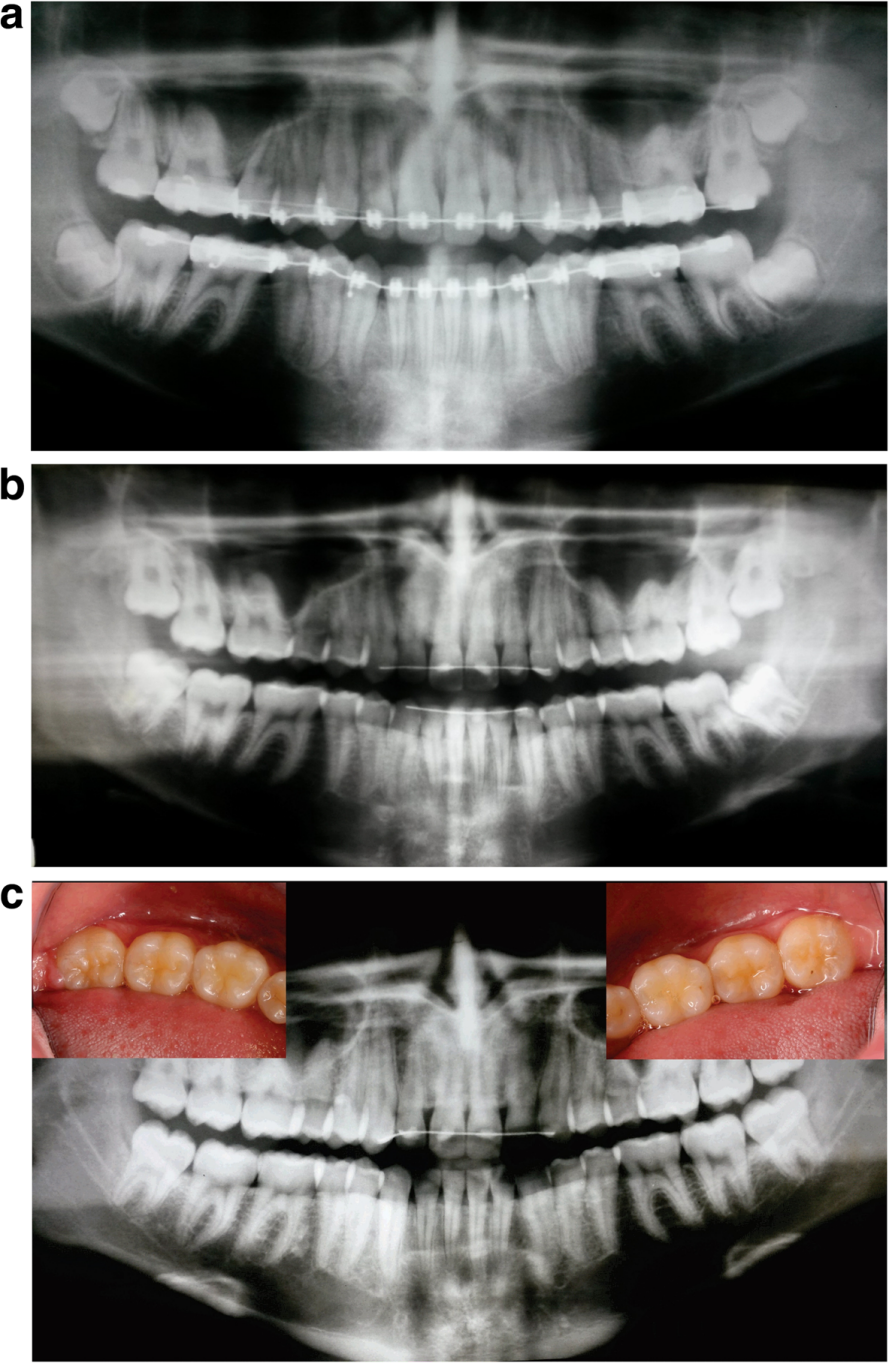
Panoramic radiographs of patient G just before bracket removal (T1, 14 years—**a**), 4 years after orthodontic treatment (18 years—**b**) and after complete eruption of the lower third molars (T2, 22 years—**c**). For this patient, OMFSs indicated third molar extraction in 82 % for the right side and 79 % for the left side, while orthodontists suggested 65 and 62 %, respectively

Intergroup comparisons disclosed that the OMFSs tended to suggest extractions more often than orthodontists (*p* < 0.0001). Orthodontists indicated extraction in 37.8 % of the cases, whereas OMFSs chose this procedure in almost half the cases (49.6 %) (Table 1, Fig. 1a–c). It was observed that whenever an orthodontist indicated extraction of a third molar, the OMFSs had a 99.2 % chance of agreement. And whenever an orthodontist decided to monitor a given case, the OMFS adopted the same approach in 81.1 % of the cases.

The questionnaire included, in addition to the basic indications, i.e., extraction or monitoring, “another conduct” option to be described by the respondents. Eight responses selected this alternative (0.8 %). However, since the responses provided no justification, all eight answers were disregarded in the statistical analysis.

All lower third molars (*n* = 34) erupted spontaneously. Therefore, the most important finding of this research was that OMFSs and orthodontists are not able to make a reliable prognosis for lower third molar eruption using a panoramic radiograph, when these teeth have spontaneous eruption. Both groups of specialists tend to over- indicate extractions, when a third molar erupted spontaneously, mainly OMFSs (Table 1).

## Discussion

When indicating extraction of third molars, dentists should have a justifiable reason, one that takes into account future treatment planning from an orthodontic, surgical, periodontal, and/or prosthetic point of view [11]. At the same time, a cost/benefit analysis should be carried out to justify the prophylactic removal of third molars, which should only be indicated with the purpose of preventing cases that involve pathological processes such as root resorption or caries in the second molar, cysts, and pericoronaritis [3, 4, 14, 15].

The most relevant finding of this study was the inability of orthodontists and OMFSs to predict third molar prognosis when these teeth erupted spontaneously. Indication was given in 37.8 % of cases by orthodontists and in 49.58 % of cases by OMFSs. These findings corroborate indication by other authors [16, 17] who—in the absence of reliable predictors—suggested that ideally third molars should be monitored through periodic evaluations. The risk of developing diseases was the key motivator leading orthodontists and OMFs to indicate third molar extractions in our findings. This concept seems to be adopted in several countries [6, 16, 17]. General dentists in the USA recommend removal of third molars in 59 % of cases [6]. A comparative analysis between the opinion of orthodontists and OMFSs, as regards to the role of third molars, found that 56.9 % of OMFSs “often” or “sometimes” recommend prophylactic removal of third molars, while 64.4 % of orthodontists “rarely” or “never” make this recommendation, underscoring a significant disagreement between these two specialists [3]. In this study, although both professionals showed significant agreement in their opinions (Kappa = 0.76), OMFSs tended to recommend more extractions than orthodontists (*p* < 0.0001). These findings support that orthodontists are more conservative than OMFSs and general dentists.

A survey comparing the views of clinicians and surgeons about the prophylactic removal of third molars conducted in Wales and Sweden found in the latter country a higher rate of third molar removal [5]. The authors explained the results by stating that in Wales, a protocol was developed which provides guidelines to decide whether or not to extract third molars, whereas such protocol is not widely accepted in Sweden. Arguably, the large number of third molar extractions currently performed is due to the lack of a protocol containing criteria that should be examined prior to recommending extraction. Despite the fact that the agreement for the responses given by the two groups of specialists was remarkable, the large number of extraction indications shows that the criteria used to support this decision must be reviewed.

An improvement in third molar position is observed in patients treated with premolar extractions [18–22]; however, when third molars are excessively tipped, they may remain impacted even if there is enough retromolar space [23]. If the probability of spontaneous eruption of third molars increases when premolars are extracted, it is likely that the reliability of prognosis is worse when these cases are assessed by orthodontists and surgeons. Thus, it seems necessary to evaluate the ability of the clinician to predict third molar eruption when orthodontic treatment involves premolar extractions.

Scientific evidence has shown that positional changes and eruption of lower third molar are unpredictable phenomena, whether in children and adolescents [8, 23] or even young adults [24]. Mandibular third molars at, or near to, the the occlusal plane and exhibiting vertical inclination were considered at highest risk for developing pericoronitis. Such third molars can be given high priority for prophylactic care due to the possibility of severe consequences of acute pericoronitis [25]. Furthermore, a higher incidence of dentigerous cysts may be associated with radiographically normal impacted lower third molar teeth [26]. Thus, prophylactic extractions of normal impacted lower third molars can be a treatment option even considering the risk of TMJ disorders [27]. The retrospective nature of this study increases the possibility of bias. A prospective follow-up study including different impaction severity and treatment outcomes should be considered, not only on an orthodontic perspective but also examining surgical complications after third molar removal [28].

## Conclusions

- Despite a remarkable agreement for third molar prognosis, oral/maxillofacial surgeons and orthodontists were unable to predict third molars eruption by examining a single panoramic radiograph. Both indicated extractions for a considerable number of spontaneously erupted asymptomatic teeth, mainly oral surgeons.
- This paper encourages clinicians to re-evaluate their view on third molar extractions based on suggested radiograph guidelines. Other diagnostic methods that are also indicated for third molar eruption prediction should be investigated, such as longitudinal radiographs or 3D images.
